# Effective CRISPR/Cas9-based nucleotide editing in zebrafish to model human genetic cardiovascular disorders

**DOI:** 10.1242/dmm.035469

**Published:** 2018-10-18

**Authors:** Federico Tessadori, Helen I. Roessler, Sanne M. C. Savelberg, Sonja Chocron, Sarah M. Kamel, Karen J. Duran, Mieke M. van Haelst, Gijs van Haaften, Jeroen Bakkers

**Affiliations:** 1 Hubrecht Institute-KNAW and UMC Utrecht, 3584 CT Utrecht, the Netherlands; 2 Department of Genetics, Center for Molecular Medicine, University Medical Center Utrecht, 3584 CX Utrecht, the Netherlands; 3 Department of Clinical Genetics, Amsterdam Medical Center, University of Amsterdam, 1105 AZ Amsterdam, the Netherlands; 4 Department of Clinical Genetics, Free University Medical Center, 1018 HV Amsterdam, the Netherlands; 5 Department of Medical Physiology, Division of Heart and Lungs, University Medical Center Utrecht, 3584 CX Utrecht, the Netherlands

**Keywords:** CRISPR/Cas9, Zebrafish, Genome editing, Point mutation, Cantú syndrome, *KCNJ8*, *ABCC9*

## Abstract

The zebrafish (*Danio rerio*) has become a popular vertebrate model organism to study organ formation and function due to its optical clarity and rapid embryonic development. The use of genetically modified zebrafish has also allowed identification of new putative therapeutic drugs. So far, most studies have relied on broad overexpression of transgenes harboring patient-derived mutations or loss-of-function mutants, which incompletely model the human disease allele in terms of expression levels or cell-type specificity of the endogenous gene of interest. Most human genetically inherited conditions are caused by alleles carrying single nucleotide changes resulting in altered gene function. Introduction of such point mutations in the zebrafish genome would be a prerequisite to recapitulate human disease but remains challenging to this day. We present an effective approach to introduce small nucleotide changes in the zebrafish genome. We generated four different knock-in lines carrying distinct human cardiovascular-disorder-causing missense mutations in their zebrafish orthologous genes by combining CRISPR/Cas9 with a short template oligonucleotide. Three of these lines carry gain-of-function mutations in genes encoding the pore-forming (Kir6.1, *KCNJ8*) and regulatory (SUR2, *ABCC9*) subunits of an ATP-sensitive potassium channel (K_ATP_) linked to Cantú syndrome (CS). Our heterozygous zebrafish knock-in lines display significantly enlarged ventricles with enhanced cardiac output and contractile function, and distinct cerebral vasodilation, demonstrating the causality of the introduced mutations for CS. These results demonstrate that introducing patient alleles in their zebrafish orthologs promises a broad application for modeling human genetic diseases, paving the way for new therapeutic strategies using this model organism.

## Abstract (precis)

**Summary:** By using a single-stranded DNA oligonucleotide template in combination with CRISPR/Cas9 in zebrafish, the authors achieved effective germline-transmissible introduction of patient-specific single-nucleotide changes related to cardiovascular disease.

## INTRODUCTION

Cardiovascular disorders are multifactorial conditions in which genetics plays an important role in pathogenicity. For example, mutations in >50 genes have been associated with the development of dilated cardiomyopathy (DCM), a condition characterized by dilatation and impaired contraction of heart muscle, and the most common form of non-ischemic cardiomyopathy worldwide (Cahill et al., 2013). For instance, DCM can be caused by an in-frame deletion in the phospholamban (*PLN*) gene (*PLN* R14del) (van der Zwaag et al., 2012). Cardiovascular disorders can also be part of rare genetic syndromes: Cantú syndrome (CS), an autosomal dominant condition, is characterized by congenital hypertrichosis, distinctive facial features and extensive cardiovascular abnormalities, including an enlarged, hypercontractile heart, pericardial effusions and diffusely dilated and tortuous blood vessels (Grange et al., 2006; Levin et al., 2016) (Table S1). Gain-of-function (GOF) missense mutations in genes encoding the pore-forming (Kir6.1, *KCNJ8*) and regulatory (SUR2, *ABCC9*) subunits of the predominantly cardiovascular isoforms of an ATP-sensitive potassium channel (K_ATP_) have been identified in CS patients (Harakalova et al., 2012; van Bon et al., 2012; Brownstein et al., 2013; Cooper et al., 2014). How these missense mutations contribute to the pathogenesis in CS is poorly understood. To improve our knowledge of gene-phenotype relations and to develop an *in vivo* model that can be used to screen or test potential therapeutic strategies, we set out to generate zebrafish models incorporating the underlying genetic cause of the diseases.

## RESULTS

**Fig. 1.**
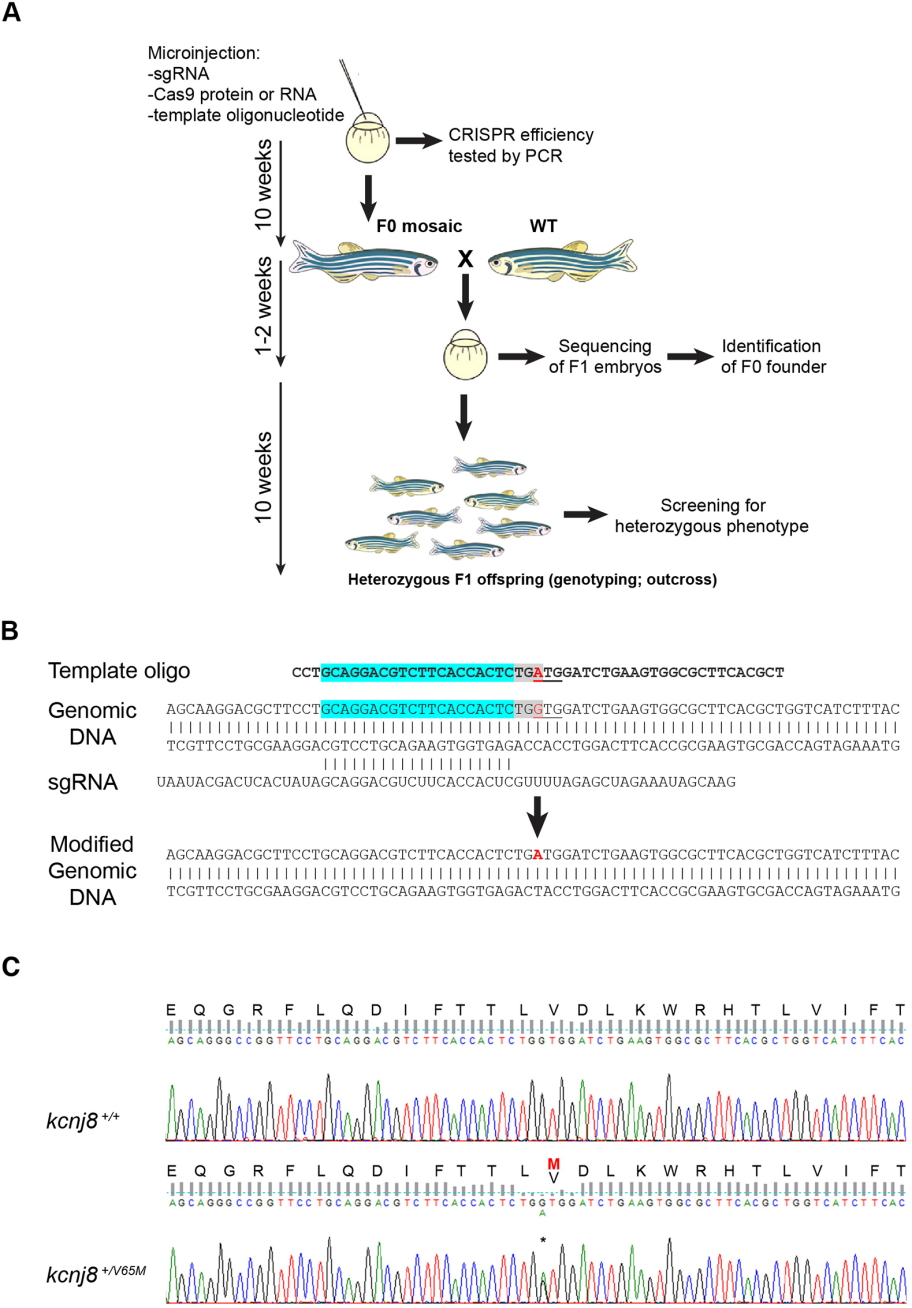
**Generation of patient-specific KI lines in zebrafish.** (A) Stepwise procedure followed to establish the KI lines described in this study with corresponding minimal timeline for each step. (B) Schematics showing the targeted genomic sequence for the introduction of the c.A193G substitution resulting in the p.V65M in the *kcnj8* genomic sequence. The PAM sequence is highlighted in gray, the specific section of the sgRNA is highlighted in cyan and the modified codon is underlined. Red text indicates substituted nucleotide. Note that in this case the substitution is located on the PAM sequence. (C) Sequencing traces for wild-type and heterozygous *kcnj8*^+/V65M^ zebrafish. Asterisk denotes the substituted nucleotide in the heterozygous *kcnj8*^+/V65M^ sequencing trace.

To introduce specific mutations in the zebrafish *abcc9*, *kcnj8* or *pln* genes, we designed CRISPR/Cas9 single-guide RNAs (sgRNAs) following previously published guidelines (Gagnon et al., 2014) and designed the sgRNAs according to the online tool ChopChop (http://chopchop.cbu.uib.no/; Fig. 1A; Fig. S1). Additionally, we provided a single-stranded 50 bp DNA template (Bedell et al., 2012) encompassing not only the site of the mutation that was being introduced (single base pair changes in the case of CS, deletion of three base pairs for *pln*), but also the protospacer adjacent motif (PAM) sequence targeted by the sgRNA (Fig. 1B; Fig. S2). Since it has been demonstrated that Cas9 cleavage occurs around 3 bp upstream of the PAM sequence (Jinek et al., 2012; Hwang et al., 2013), we selected specific mutations as close as possible to a potential PAM sequence, as we reasoned that this would maximize efficiency of the introduction of the chosen mutation. For all four mutations presented in this study, this was possible within 4 bp of a potential PAM sequence. Furthermore, the template oligonucleotide was not only designed to introduce the patient-specific mutation(s), but also carried silent mutations that removed the PAM recognition sequence to prevent targeting of Cas9 after introduction of the mutation in the genome. For Pln*_*R14Del, the mutation introduced a *Bgl*I restriction site, which was used for genotyping purposes (Fig. S2). We provided Cas9 nuclease to the microinjection mixture as mRNA or purified protein (Table 1).

**Table 1.**
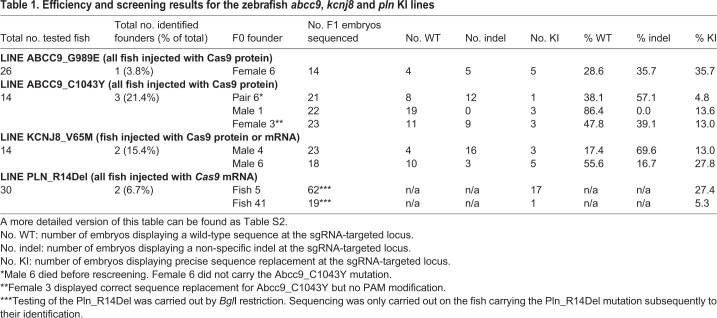
**Efficiency and screening results for the zebrafish *abcc9*, *kcnj8* and *pln* KI lines**

Figure 1 presents an overview of the steps to generate knock-in (KI)-specific zebrafish lines. After microinjection of the CRISPR/Cas9 without oligo template was carried out in the first cell of zebrafish embryos, these were let to grow until 24 hours post-fertilization (hpf). Healthy microinjected embryos were assayed for efficiency of the guide RNA, using an adapted method for detecting simple sequence-length polymorphisms (SSLPs; Knapik et al., 1998; Fig. S3). For the sgRNAs we tested, we observed relatively high indel (insertion/deletion) introduction efficiencies, as they varied from between 71.4 and 100% [*ABCC9* G989E: 21/28 (75%); *ABCC9* C1043Y: 21/28 (75%); *KCNJ8* V65M: 28/28 (100%); *PLN* R14Del: 20/28 (71.4%); Fig. S3). For all tested sgRNAs for CS and *PLN* R14Del, we proceeded to microinjections with addition of template oligonucleotide and tested their incorporation by Sanger sequencing or, for *PLN* R14Del, by using a *Bgl*I restriction site (Figs S2 and S3). Microinjected siblings (F0) of the tested embryos were grown to adulthood, including embryos microinjected without the template oligonucleotide, in order to obtain adult mosaic zebrafish founders (F0) for the KI and knockout (KO) lines. To determine successful introduction of indels or specific mutations, we carried out sequencing of the progeny of F0 fish (Fig. 1C; Fig. S4). For KO lines, we obtained positive results in 67-100% of the analyzed larvae (not shown). For KI lines, mosaic founders carried various non-specific indels or unmodified genomic loci in addition to precise introduction of the mutated sequences, the efficiency of which ranged from 3.8 to 21.4% (Table 1; Tables S1 and S2). We also checked for mutational events at genomic sites displaying sequence similarity with the used sgRNAs, and did not observe any off-target effect (Fig. S5). For all lines, sequencing analysis of the progeny of F1 fish after outcross to a WT line confirmed that truncating indels or specific nucleotide mutations were introduced and segregate, as expected, in a Mendelian fashion. As displayed in Fig. 1, our approach allows generation of stable KI heterozygous zebrafish lines in as little as 22 weeks. Although somewhat costlier and less efficient than the generation of KO mutants (Fig. S6), generation of KI lines could be achieved in the same time frame as the corresponding KO lines, which were also generated for phenotypic comparison.

**Fig. 2.**
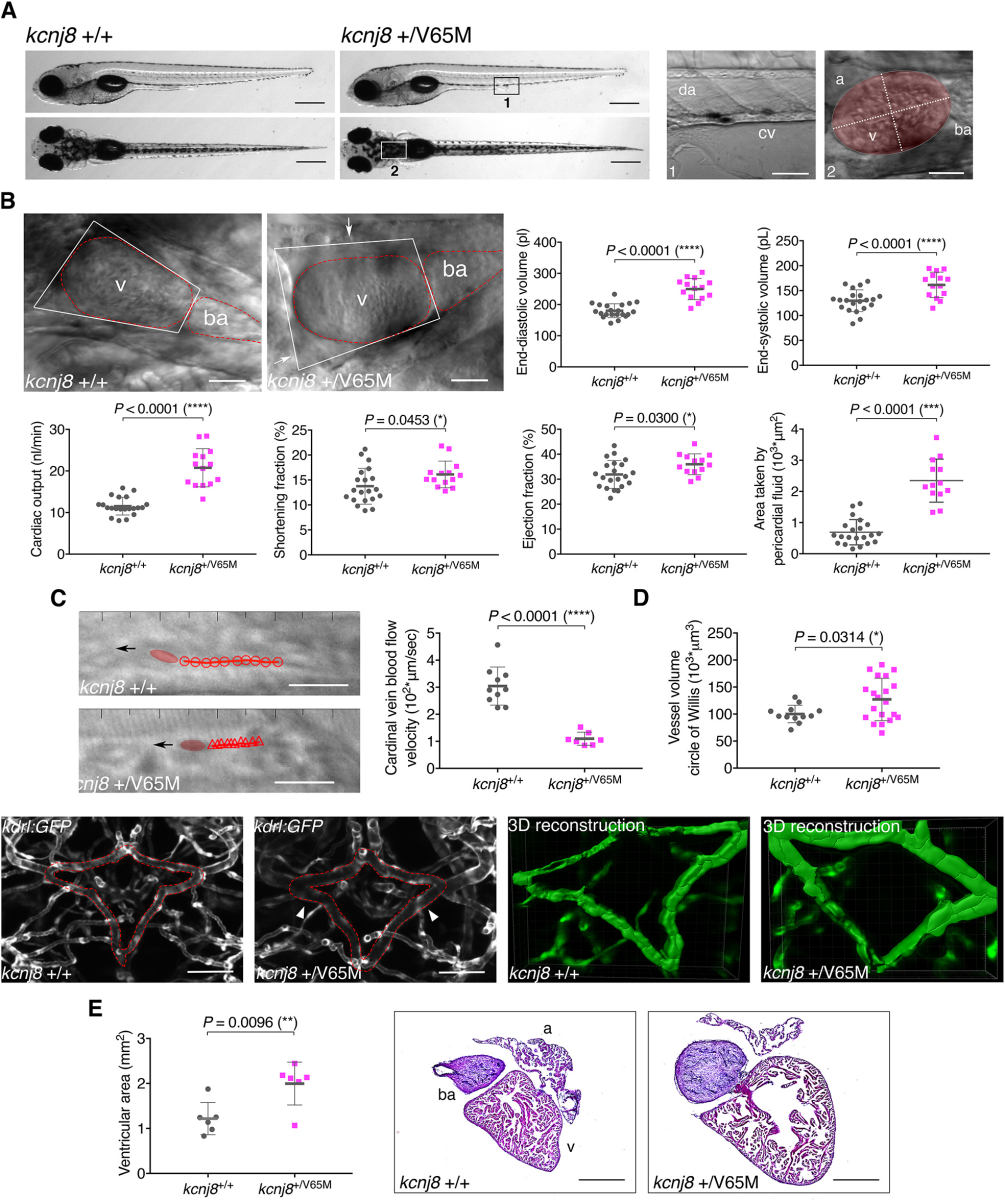
**Heterozygous *kcnj8*^+/V65M^ mutation induces CS-related cardiac anomalies and cerebral vasodilation in zebrafish.** (A) Representative images illustrating the morphology of 5 dpf wild-type and *kcnj8*^+/V65M^ mutants as seen from a left lateral (top) and dorsal (bottom) view. Boxes designate imaged areas that were used to assess cardiac function: the cardinal vein (1) and the heart (2). The ventricular area of the heart is highlighted, with the long axis and short axis of the ventricle indicated by dashed lines. a, atrium; ba, bulbous ateriosus; cv, cardinal vein; da, dorsal aorta; v, ventricle. (B) Quantification of cardiac function using individual characteristic confocal sections from a time series of the embryonic cardiac cycle at 5 dpf. Pericardial edema was quantified by measuring pericardial area using striking morphological landmarks, indicated by white boxes. Ventricular area was subtracted. Arrows show accumulation of fluid in *kcnj8*^+/V65M^ mutants. Dotted red lines indicate ventricle (v) and bulbous arteriosus (ba). (C) Tracking of individual red blood cells (RBCs) measuring blood flow velocity in the cardinal vein. RBCs were tracked for ten frames using ImageJ (NIH) and the plugin MTrackJ (Meijering et al., 2012). One representative image of each genotype is shown. Black arrow indicates the direction of RBC movement. (D) Quantification of vascular dilations in a Tg(*kdrl:GFP*) background. Representative confocal images of the circular structure comprising the BCA and PCS in wild-type and heterozygous 5 dpf fish are outlined in red. The arrowheads indicate distinct regions of vasodilation. 3D reconstruction of vascular structure in Imaris was used to calculate vessel volume. (E) Ventricular area in heterozygous *kcnj8*^+/V65M^ mutants. Representative heart histology of adult *kcnj8*^+/V^^65M^ mutants and respective wild-type siblings after H&E staining. Exemplary depiction of one WT and one *kcnj8*^+/V65M^ heart. For assessment of ventricular chamber size, tissue sections showing the largest ventricular area were selected and area was quantified using ImageJ (NIH). For all graphs, significance was determined by two-tailed unpaired Student's *t*-test or Mann–Whitney two-tailed *U*-test: *P≤0.05; **P≤0.01; ***P≤0.001; ****P≤0.0001. The black horizontal bar indicates the mean value for each condition. Sample sizes: (B) *kcnj8*^+/+^, *n*=21; *kcnj8*^+/V65M^, *n*=14; (C) *kcnj8*^+/+^, *n*=10; *kcnj8*^+/V65M^, *n*=7; (D) *kcnj8*^+/+^, *n*=12; *kcnj8*^+/V65M^, *n*=20; (E) *kcnj8*^+/+^, *n*=6; *kcnj8*^+/V65M^, *n*=6. Scale bars: (A) 1 mm (top and middle) and 50 µm (bottom); (B) 50 µm; (C) 10 μm; (D) 50 µm; (E) 500 µm. All embryos analyzed originated from group matings of adult zebrafish.

We then proceeded to systematically test the functional validity of both our heterozygous and homozygous CRISPR-generated lines. Both heterozygous and homozygous larvae were morphologically inconspicuous (Fig. 2A; Figs S7A, S10A and S11A), viable through adulthood and fertile. Thanks to its optical clarity and simple yet functionally relevant heart structure, the zebrafish embryo provides an excellent model for studying pathophysiological cardiovascular development *in vivo*. We applied live high-speed video imaging of the beating embryonic heart, cardinal vein and dorsal aorta of zebrafish to quantify cardiac function (Hoage et al., 2012) in the KI lines described in this study (Fig. 2A). In *pln^+/R14Del^* larvae, we could not detect any anomalous cardiac function (not shown). This did not come as a surprise since, in contrast to CS, DCM is not a congenital condition and may hence have no effect on embryos. We therefore continued functional characterization exclusively on CS larvae.

Remarkably, analogous to CS patients (Brownstein et al., 2013; Cooper et al., 2014; Levin et al., 2016), heterozygous *kcnj8*^+/V65M^ KI larvae showed significantly elevated mean end-diastolic (mEDV) and end-systolic (mESV) volumes with strikingly enhanced cardiac output (53%) (Fig. 2B) due to equally increased stroke volume (*P*<0.0001) at 5 days post-fertilization (dpf) (Fig. S7). Additionally, contractile function was elevated and an increased amount of pericardial edema (*P*<0.05) was observed (Fig. 2B; Figs S7 and S8B, Movies 1 and 2). A significantly reduced cardinal vein and dorsal aorta blood flow velocity (Fig. 2C, *P*<0.0001; Fig. S8C, *P*<0.0062; Movies 3 and 4) was measured in heterozygous *kcnj8^+/^*^V65M^ larvae, and can be associated with low blood pressure and diminished vascular tone reported in CS patients (Levin et al., 2016). Homozygous *kcnj8*^V65M/V65M^ embryos presented with similar features (Figs S7 and S8).

Additionally, cardiomegaly was studied in 3-month-old *kcnj8* V65M fish and respective wild-type siblings. Here, hearts were sectioned and stained with Hematoxylin and Eosin (H&E; Fig. 2E, Fig. S9A,B). For individual assessment of ventricular and atrial size, tissue sections revealing the largest chamber area were selected and area was measured. In accordance with our findings in 5 dpf larvae and in CS patients (Levin et al., 2016), heterozygous adult fish reveal a significantly enlarged ventricular chamber volume (*P*=0.0096; Fig. 2E), whereas atrial area remains similar to wild-type size (Fig. S9B). The same was observed in homozygous *kcnj8*^V65M/V65M^ mutants (Fig. S9A,B). To account for variations in heart size, overall body length was measured, revealing no difference in size between all studied genotypes (Fig. S9C).

Quantification of cardiac function in heterozygous and homozygous *abcc9* G989E KI larvae revealed a similar phenotypic spectrum (Fig. S10B-E), whereas *abcc9* C1043Y KI embryos showed no distinct CS-related cardiac pathology (Fig. S11B-E). Notably, this recapitulates the features of the only patient known with a C1043Y variant, who is reported to lack cardiomegaly as well as other cardiac features (van Bon et al., 2012). Additionally, CS presents as a strikingly heterogeneous condition with only hypertrichosis and coarse facial appearance present in all cases. Further features, such as pericardial effusions, enlarged hearts and hypercontractile hearts, are only exhibited by a certain number of cases, illustrating the fact that the penetrance of CS-associated features is inconsistent, without clear correlation to genotype. Therefore, respective phenotypical variability in zebrafish is appropriately in line with the observed inconsistent distribution of features in patients.

To characterize aberrant blood vessels, we examined the entire vasculature of live 5 dpf mutants in a Tg(*kdrl:GFP*) transgenic background for dilations and tortuosity by applying confocal microscopy. Given that CS patients predominately present with cerebral vascular anomalies (Leon Guerrero et al., 2016), we focused on the same area in all CRISPR-generated CS lines. An initial systematic examination of larvae from heterozygous *kcnj8*^+/V65M^ incrosses revealed a diffusely dilated circular structure comprising the basal communicating artery (BCA) and posterior communicating segments (PCS). This structure resembles the human circle of Willis, a prominent area of vascular anomalies in CS patients (Brownstein et al., 2013; Leon Guerrero et al., 2016). A quantification of the vascular volume of this structure identified significant (*P*<0.05) cerebral vasodilation displayed in heterozygous fish (Fig. 2D). The observed variation is in line with intra-familial variability for the different features reported in CS patients (Roessler et al., 2018). Homozygous mutants show similar vasodilation (Fig. S6D). No further abnormalities of the vasculature in whole embryos could be observed during the initial examination.

The same strategy was applied to larvae from either *abcc9*^+/G989E^ or *abcc9*^+/C1043Y^ heterozygous parents, which likewise revealed vascular abnormalities exclusively in the BCA/PCS vessel structure. Whereas both heterozygous and homozygous *abcc9* G989E KI fish showed comparable vasodilation (*P*>0.05; Fig. S10F) but no tortuosity, *abcc9* C1043Y larvae presented with tortuous but normally sized BCA and PCS (Fig. S11F).

Next to cardiovascular anomalies, *kcnj8* V65M mutants were assessed for craniofacial phenotypes by Alcian Blue staining at 5 dpf (Fig. S12), revealing no cartilage deformations in both heterozygous and homozygous larvae. Notably, the origin of craniofacial features in CS patients is not fully determined yet. For instance, acromegaloid facial appearance so far observed in all CS cases may not descend from bone deformities but rather facial swellings due to underlying edema. This possibility is supported by an observed overlapping facial and edematous phenotype in individuals with CS and individuals treated with Minoxidil, a K_ATP_ channel opener (Mehta et al., 1975; Kaler et al., 1987).

Additionally, all CRISPR-generated KI lines were assessed for macrocephaly and macrosomia, respectively – two features frequently observed in CS patients at birth. However, no difference between KI larvae and wild-type controls was observed (not shown). The absence of these non-cardiovascular CS features in the CRISPR-generated KI lines at 5 dpf may be due to the lack of bone formation at this developmental time point in zebrafish or differences between zebrafish and human physiology. Further studies are required to resolve this question.

## DISCUSSION

In the study presented here, we were successful in generating four different KI lines by introducing four distinct missense mutations causing human cardiovascular disorders. This was achieved by combining the CRISPR/Cas9 system with a specific template oligonucleotide, which allowed us to introduce the disease-causing missense mutations into the corresponding zebrafish orthologous genes. It is important to note that parameters such as a high initial efficiency of the sgRNA and the proximity of the PAM with the desired site of mutations are paramount to the success of the method (Figs S3 and S4). Indeed, our study presents successful small nucleotide changes located within 4 bp of the corresponding PAM site. This parameter, which constitutes a limitation of our approach, should become less stringent in the close future, as evolved Cas9 proteins with a broader range of PAM requirements for targeting DNA sequences have very recently been published (Hu et al., 2018). These promise improved capacity for the CRISPR/Cas9 system to model patient-specific mutations in animal or *in vitro* models.

Our work demonstrates that CS-associated variants in both *kcnj8* and *abcc9* lead to a remarkable combination of cardiovascular anomalies in zebrafish, such as enlarged, hypercontractile ventricles with increased cardiac output, elevated occurrence of pericardial edema, reduced blood vein flow velocity, and cerebral vasodilation and tortuosity. Importantly, we observed that larvae heterozygous for *kcnj8* or *abcc9* mutations display these anomalies, which is in good agreement with the inheritance mode of CS (autosomal dominant). Animals with the *kcnj8*^+/V65M^ allele displayed stronger phenotypes than both *abcc9* KI lines. Notably, clinical hallmarks in patients harboring a missense mutation in *KCNJ8* are generally considered to be more severe than in patients with an *ABCC9* mutation (Brownstein et al., 2013; Cooper et al., 2014). Hence, our novel zebrafish models recapitulate characteristic hallmarks clinically observed in CS patients, and thereby confirm the causality of *KCNJ8* and *ABCC9* mutations for CS (Grange et al., 2006; Leon Guerrero et al., 2016; Levin et al., 2016). The presence of CS key features in all CRISPR-generated KI lines further demonstrates that initial molecular consequences must originate from tissues expressing both Kir6.1 and SUR2 subunits.

K_ATP_ channels are hetero-octameric complexes, with *ABCC9* (SUR2) and *KCNJ8* (Kir6.1) prominently expressed in cardiomyocytes, vascular smooth muscle and vascular endothelial cells (Flagg et al., 2010), suggesting that cardiovascular features may predominate in CS (Armstrong et al., 2016). Naturally, gain of K_ATP_ channel function would be expected to cause reduced contractility and vasodilation in blood vessels and action potential shortening resulting in reduced cardiac contractility and output in the heart (Nichols, 2006). Although our vascular findings are consistent with these expected consequences of K_ATP_ GOF, both *kcnj8*^+/V65M^ and *abcc9*^+/G989E^ KI fish manifest a high output state with enhanced cardiac function, a pathophysiology opposite to the prediction. In mice with constitutive or tamoxifen-induced cardiac-specific Kir6.1 GOF subunit expression, this counter-observation can be explained by a compensatory increase in basal L-type Ca^2+^ current, paralleled by changes in phosphorylation of the pore-forming α_1_ subunit of the cardiac voltage-gated calcium channel Cav1.2 resulting in remodeling of cardiac excitation-contraction coupling, and hence hypercontractility and high cardiac output (Levin et al., 2016). Further studies are necessary to confirm the alteration of this mechanism in the described CS zebrafish mutants.

With cardiac output being directly proportional to blood pressure, we additionally propose that the observed high-output state compensates for hypotension due to vasodilation in CS patients. At the moment, there is no treatment for CS available. However, the CS K_ATP_ channel can be pharmaceutically targeted by several approved drugs, such as second-generation sulfonylureas, which are already used in clinical setups to inhibit GOF in the pancreatic K_ATP_ isoforms involved in neonatal diabetes mellitus (Ashcroft, 2010; McClenaghan et al., 2018). The successful validation of our CS KI zebrafish may provide the opportunity of phenotype-based screening to test the efficiency of these and further potential therapeutic compounds. Consequently, future studies in our CS zebrafish models will help to shed light on underlying molecular mechanisms and contribute to the development of a treatment for the disorder, insights that may improve understanding and therapeutic management of CS.

In conclusion, we have improved a CRISPR/Cas9-based single-nucleotide-editing approach with which we established novel zebrafish KI lines that closely model cardiovascular features of human disease. Beyond the application illustrated here, our approach offers a versatile tool to edit the zebrafish genome at the nucleotide level and provide broad possibilities for modeling of patient-specific mutations.

## MATERIALS AND METHODS

### Fish maintenance and preparation

All animal experiments were conducted under the guidelines of the animal welfare committee of the Royal Netherlands Academy of Arts and Sciences (KNAW). Adult zebrafish (*Danio rerio*) were maintained and embryos raised and staged as previously described (Westerfield, 1993). The zebrafish lines used in this study were Tübingen longfin (wild type) and Tg(*kdrl:GFP*) (Choi et al., 2007).

Adding phenylthiourea (PTU) at a concentration of 0.003% (v/v) to the E3 embryonic raising medium at the Prim-5 stage (about 24 hpf) blocked pigmentation for high-speed imaging and confocal analysis.

### *Cas9* mRNA expression plasmid and Cas9 protein

Plasmid pCS2-nCas9n (Addgene #47929) was used as template for synthesis of capped mRNA (see below for a more detailed description). Cas9 nuclease protein was purchased at NEB (catalog number NEBM0386M).

### sgRNA and template oligonucleotide

Design and synthesis of sgRNAs used in this study were fundamentally carried out following the guidelines given in previous literature (Gagnon et al., 2014; Chari et al., 2015), with minor modifications. All single-stranded DNA oligonucleotides (sgRNA template, constant oligonucleotide, template oligonucleotide) were purchased at Integrated DNA Technologies (IDT) as standard desalted same-day oligos. A table detailing all ssDNAs used in this study can be found in Fig. S2.

### sgRNA and *Cas9* mRNA synthesis

Synthesis of the sgRNA specific to each mutation was carried out using the Ambion MEGAscript T7 or SP6 kits (Ambion). *Cas9* mRNA was synthesized by linearizing pCS2-nCas9n (Addgene #47929) with the restriction enzyme *Not*I and subsequently using the linearized plasmid as template for the SP6 mMessage Machine kit (Ambion) to produce capped mRNA. Purification of the *in vitro* synthesized mRNA was achieved with the RNeasy Mini Kit (Qiagen).

### Microinjection in zebrafish embryos

One-cell-stage zebrafish embryos were microinjected with an injection mixture consisting of (final concentrations): 150 ng/μl nuclear Cas9 (*nCas9*) mRNA or 5 μM nCas9 protein, 20-40 ng/μl sgRNA, 10% (v/v) Phenol Red and 25 ng/μl template oligo. nCas9 mRNA and protein microinjections were carried in the cell. At 24 hpf, genomic DNA was extracted from microinjected, healthy embryos and sequenced for assessment of CRISPR/Cas9 introduction of genomic editing.

### PCR and electrophoresis-based genotyping

PCR primers encompassing the CRISPR sites (see Fig. S2 for primer sequences) were used to produce PCR products of approximately 250 bp. The PCR products were then loaded and migrated by electrophoresis on a high percentage (2-4%) Tris-borate-EDTA (TBE) agarose gel supplemented with ethidium bromide. The minimal differences in migration speed of the PCR products caused by the CRISPR/Cas9 genome editing can be visualized on the electrophoresis gel. Deletions and insertions can be easily visualized as distinct bands or smears on a 4% agarose gel after sufficient migration time (Fig. S3). Restriction for genotyping of PLN_R14Del was carried out with *Bgl*I (NEB).

### Sequencing

CRISPR site-specific PCR primers were used to amplify the flanking genomic regions of the CRISPR target sites. Taq Gold polymerase (Applied Biosystems, Thermo Fisher Scientific, Waltham, MA, USA) was used [1 µl isolated DNA, 2.5 mM MgCl_2_, 0.2 mM deoxynuclotides (dNPTs), 1× Taq buffer, 20 pmol of both primers, 1 U Taq polymerase] in combination with a touch down PCR program: initial denaturation at 95°C for 5 min, followed by 15 cycles of denaturation at 95°C for 30 s, annealing at 65°C to 57.5°C for 30 s by decreasing 0.5°C steps cycle-wise (‘touchdown’ PCR) and extension at 72°C for 1 min, followed by 27 cycles of denaturation at 95°C for 30 s, annealing at 58°C for 30 s and extension at 72°C for 1 min, followed by a final extension step at 72°C for 7 min and infinite hold at 8°C. Amplicons of KO fish were visualized on a 3% agarose gel and specific KI mutations were confirmed by Sanger sequencing.

### *In vivo* high-speed imaging

Image acquisition was conducted using a Hamamatsu C9300-221 high-speed CCD camera (Hamamatsu Photonics) at 150 frames per second (fps) mounted on a Leica DM IRBE inverted microscope (Leica Microsystems) using Hokawo 2.1 imaging software (Hamamatsu Photonics). Image analysis was subsequently carried out with ImageJ (http://rsbweb.nih.gov/ij/, last accessed November 2017). For analysis of cardiac function, adult zebrafish heterozygous for the respective mutations in *kcnj8* or *abcc9* were interbred, and genotyped by Sanger sequencing post-imaging. High-speed brightfield image sequences of the embryonic zebrafish heart were acquired for zebrafish at 5 dpf using a 20-fold magnification. Zebrafish were anesthetized in 16 mg/ml tricaine (MS222; Sigma-Aldrich) in E3 medium and mounted in dorsal position in small microscopic chambers filled with 0.25% (w/v) agarose (Sigma-Aldrich) prepared in the same concentration of anesthetic. Zebrafish hearts were imaged for 10 s (approximately 30 cardiac cycles) at 28±0.3°C.

### Measurement of ventricular volume, cardiac output, shortening fraction, fractional area change and ejection fraction

The time interval between three heartbeats was measured and the heart rate (bpm) was calculated. Images from high-speed movies were used to outline the perimeter of the ventricle. Measurement analysis was carried out using the ‘fit-to-ellipse’ algorithm, which worked on calculating the center of mass and subsequently the best-fitting ellipse. The long axis length (a) and short axis length (b) at diastole and systole were determined and used to calculate ventricular end-systolic (ESV) and diastolic (EDV) volumes applying the formula: V=4/3π(b/2)^2^(a/2). The stroke volume (SV) was calculated as the difference between three ventricular EDVs and ESVs. Cardiac output was obtained by multiplying the heart rate with stroke volume. The percent shortening fraction (SF) was calculated using the formula: SF(%)=(length at diastole−length at systole)/(length at diastole)×100. Ejection fraction (EF) can be calculated with the following formula: EF(%)=(SV/EDV)×100. Fractional area change (FAC) was calculated as follows: FAC(%)=(area at diastole–area at systole)/(area at diastole)×100. Every cardiac parameter was calculated in triplicate.

### Blood flow velocity

Zebrafish were mounted in lateral position at 5 dpf and the region behind the cloaca showing both cardinal vein and dorsal aorta was imaged for 10 s at 28±0.5°C. Blood flow velocity was calculated by assessing frame-by-frame motion of three single erythrocytes per fish (at least seven fish per group) determined from high-speed images to assess mean erythrocyte cell velocity (µm/second) using ImageJ. Cardinal blood flow velocity was measured over ten frames at a video frame rate of 150 fps as non-pulsatory venous flow allows frame-by-frame analysis. In contrast, dorsal aorta blood flow velocity was analyzed over the whole imaged area to account for pulsatory blood flow.

### Quantification of pericardial edema

Images at cardiac diastole with focus on ventricular perimeter were taken from high-speed movies to outline pericardial area. Striking morphological landmarks, such as the ventriculo-bulbar valves and the inner pericardial wall, were used to obtain the same area in every imaged embryo. In order to correct for possible enlarged ventricular size in mutants, ventricular area was subtracted.

### Heart extraction and H&E staining

Zebrafish hearts were dissected and fixed in 4% paraformaldehyde in PBS at 4°C overnight and subjected to paraffin embedding and sectioning at 10 μm intervals. Heart sections were stained with H&E following standard procedures. Six fish were used for each genotype.

### Quantification of heart chamber size

To assess ventricular and atrial chamber size, tissue sections showing the largest ventricular and atrial area were selected and area was measured using ImageJ (NIH).

### *In vivo* confocal microscopy and image quantification

Confocal imaging of cerebrovasculture was carried out on a Leica SPE confocal microscope (Leica Microsystems) using a 10× and 20× objective. Adult zebrafish heterozygous for *kcnj8* V65M, *abcc9* G989E and *abcc9* C1043Y and carrying the *kdrl:GFP* transgene were interbred, and genotype identified by Sanger sequencing post-imaging. Zebrafish at 5 dpf were anesthetized with 16 mg/ml tricaine and mounted in dorsal position in 0.25% agarose. 3D quantification of cerebral vasculature was carried out with Imaris software (Bitplane, Oxford Instruments). A Leica SPE confocal system (Leica Microsystems) was used to generate confocal stacks of approximately 250 μm with a slice interval of 1 μm under identical imaging conditions for all samples in an experiment. Stacks were aligned and reconstructed into a 3D volume using Imaris. The volume of the structure resembling the human circle of Willis was measured after eliminating any interfering vessels.

### Whole-embryo brightfield imaging

*In vivo* phenotypic assessment for whole-embryo imaging were carried out on a Leica M165FC stereomicroscope (Leica Microsystems) with transmitted light. Images were captured with a DFC420 digital microscope camera (Leica Microsystems).

### Cartilage staining (Alcian Blue)

Zebrafish larvae at 5 dpf were incubated overnight at 4°C in fixative solution (76% ethanol; 20% acetic acid; 4% formaldehyde supplemented with 15mg Alcian Blue). Subsequently, larvae were washed in 70-100% ethanol, briefly transferred to 100% methanol and finally imaged in Murray's solution (v/v: 2:1 benzylbenzoate:benzylalcohol).

### Measurement of macrocephaly and body length

To assess macrocephaly in heterozygous and homozygous mutants, whole-embryo brightfield images were applied to measure the distance between the convex tip of the eyes (interorbital distance) in 5 dpf larvae using ImageJ (NIH; https://imagej.nih.gov/ij/). Overall, larval and adult body length was measured from the tip of the head to the end of the trunk (before the caudal fin)

### Statistical analysis

Sample size was not predetermined by statistical analysis. In all experiments involving zebrafish embryos, selection was random for scoring. Exact numbers of analyzed embryos are reported at relevant locations in the main text or the supplementary information. Statistical analysis was carried out with Prism (GraphPad). Normal distribution of the data sets was confirmed by D'Agostino–Pearson omnibus normality test. Significance values were calculated using an unpaired Student's *t*-test throughout the manuscript. All values are expressed as mean±s.d.
